# Progress in Surgery of the Liver and Gall Bladder

**Published:** 1903-10-24

**Authors:** 


### Progress in Surgery of the Liver and Gall Bladder.

*(Concluded from page 51.)*

**Cholecystectomy**.—Park[7] points out that the appendix and gall-bladder closely resemble each other in their pathological conditions. He holds that the diseased and troublesome gall-bladder, like the diseased and troublesome appendix, should be removed, and that surgeons should now recognise cholecystectomy as an ideal operation corresponding to appendicectomy. The author maintains that one operation is not more dangerous than the other, and is equally satisfactory. It is fair to maintain, he states, that when the gall-bladder is occluded, and contains calculi and more or less inspissated or altered mucus, it has ceased to perform its proper function, and is a source of offence. Moynihan[8] says cholecystectomy is called for in the following conditions:—1. In injuries of the gall-bladder, rupture, stabs, or bullet wounds. 2. In gangrene of the gall-bladder. 3. In phlegmonous cholecystitis. 4. In membranous cholecystitis. 5. In chronic cholecystitis, with dense thickening of the walls of the gall-bladder and cystic duct, with or without stenosis of the cystic duct, and in chronic cholecystitis, when the gall-bladder is shrivelled and puckered, and universally adherent. In such cases it is no longer a receptacle for the bile. 6. In distension of the gall-bladder, by dropsy or empyema, due to blocking of the cystic duct by calculus, stricture, growth, or external inflammatory deposits; or in cases of mucous fistula following operations for these conditions. 7. In cases of fistula between the gall-bladder or the cystic duct on the one hand and the stomach, duodenum, or the colon on the other. 8. In multiple ulcerations of the gall-bladder or cystic duct, when gall-stones have eroded their way through the walls into the liver, the duodenum, or other protective adhesions. 9. In primary carcinoma of the gall-bladder. The gall-bladder should never be removed unless the surgeon is convinced that the common duct is patent. Mayo has suggested a modification of cholecystectomy, which is said to be more readily performed, and is therefore, a less serious procedure. It is performed in cases of permanent obstruction of the cystic duct, the common duct being permeable, and consists in the removal of the entire mucous membrane of the gall-bladder. The mucous membrane at the fundus is separated with some difficulty, but at and near the neck it strips away easily. After its removal the other coats are stitched to the upper angle of the wound. In the performance of complete cholecystectomy the peritoneal investment of the gall-bladder is cut through along the line of its reflexion on to the liver, and the gall-bladder is quickly stripped from the liver and grasped in the hand so as to exert a gentle traction upon the cystic duct. A cuff of peritoneum is then reflected from the neck of the gall-bladder and cystic duct, the duct tied with catgut and the gall-bladder cut away, and the peritoneal cuff is then replaced and sutured over the end of the stump as in appendicectomy.

**Hepatic Cirrhosis.**—Leport[9] has collected a series of 53 cases treated by Talma's operation of fixing the omentum to the abdominal wall. In 20 there was a complete cure, and in 22 there was only slight improvement or a fatal result. The operation being in itself simple, there are practically no contra-indications, and where other methods of dealing with cirrhotic ascites have failed it should certainly be tried. Greenhough[10] summarises the subject as follows: 1. The condition known as biliary cirrhosis, with enlarged liver, jaundice, and fever without ascites, is accompanied in a certain proportion of cases by an infection of the bile-ducts. The drainage of the bile-ducts by cholecystostomy is a proper operation for the relief of this condition when evidence of infection is present and symptomatic treatment has failed to effect relief. 2. Of 105 cases of liver cirrhosis which presented the symptoms of ascites, 42 per cent. were improved and 58 per cent. not improved by Talma's operation or one of its modifications. The mortality within thirty days was 29·5 per cent. Nine cases were improved in health two years after the operation. 3. The nine cases of continued relief presented no marked difference from the general character of all the cases. 4. The cases in which the liver was enlarged gave a lower mortality and a higher percentage of improvement than cases of atrophic liver. 5. Cases of suture of the omentum between the layers of the abdominal wall gave a lower mortality and a slightly higher percentage of improvement than cases where only peritoneal surfaces were brought in contact. 6. Drainage increases the danger of septic infection and peritonitis and is to be avoided. If necessary, tapping may be done after the operation. 7. The presence of adhesions or perihepatitis is of good prognostic import as regards the success of the operation. 8. The number of tappings before operation and the presence of œdema of the feet and legs are of less prognostic importance than the general condition of the patient, the size of the liver, and the functional activity of the liver cells. 9. Talma's operation or one of its modifications is of proved benefit in a limited number of cases of liver cirrhosis, primarily for the relief of ascites, and secondarily for the relief of other symptoms of portal congestion. 10. The dangers attending the operation are mainly due to the weakened resistance of the patient rather than to the operation itself. The selection of cases suitable for operation demands more judgment than has hitherto been exercised. 11. The operation is not indicated in cases of ascites due to causes other than cirrhosis of the liver. 12. The operation is contra-indicated in the presence of renal or cardiac disease and when good evidence does not exist that sufficient functional liver tissue remains to maintain life. It is also contra-indicated when complications exist sufficient in themselves to make the result of operation uncertain.

[7] Brit. Med. Jour., Jan. 10, 1903, Epitome No. 18. [8] Ibid., Jan. 24, 1903, p. 186. [9] Ibid., Jan. 17, 1903, Epitome No. 39. [10] Amer. Jour. Med. Sci., Dec. 1902, p. 980.

